# Landscape structure as a driver of eco-evolution in host–parasite systems

**DOI:** 10.1093/evlett/qraf003

**Published:** 2025-04-01

**Authors:** Jhelam N Deshpande, Vasilis Dakos, Oliver Kaltz, Emanuel A Fronhofer

**Affiliations:** Institut des Sciences de l’Evolution - Montpellier (ISEM), Université de Montpellier, CNRS, IRD, Montpellier 34095, France; Institut des Sciences de l’Evolution - Montpellier (ISEM), Université de Montpellier, CNRS, IRD, Montpellier 34095, France; Institut des Sciences de l’Evolution - Montpellier (ISEM), Université de Montpellier, CNRS, IRD, Montpellier 34095, France; Institut des Sciences de l’Evolution - Montpellier (ISEM), Université de Montpellier, CNRS, IRD, Montpellier 34095, France

**Keywords:** spatial networks, host–parasite systems, virulence evolution, landscape, epidemiology

## Abstract

Spatial network structure of biological systems drives ecology and evolution by distributing organisms and their genes. The ubiquitous host–parasite systems are no exception. However, past theoretical work has largely focused on simple spatial structures, such as grids, hampering the translation of theoretical predictions to real ecosystems. Thus, we develop an eco-evolutionary metapopulation model of host–parasite dynamics where hosts and parasites disperse through realistically complex spatial networks representing major biomes using river-like and terrestrial-like networks. We generate the testable prediction that parasite virulence, or how parasites harm their hosts, peaks at intermediate dispersal values in river-like systems while it increases with increasing dispersal in terrestrial-like systems. In river-like systems, virulence also reaches higher overall values. Moreover, we show that kin selection is the main driver of virulence evolution. Spatial networks generate characteristic patterns of parasite relatedness which drive differential virulence evolution. Finally, we show that accounting for virulence evolution allows us to predict the distribution of key epidemiological variables (e.g., parasite extinction risks) within spatial networks. Our study highlights how eco-evolutionary feedbacks can be understood in light of spatial network structure by linking network topology to classical evolutionary mechanisms such as kin selection.

## Introduction

All biological and many artificial systems are fundamentally spatially structured. Spatial structure can be represented by networks (Urban & Keitt, 2001) where, in ecology and evolution, nodes are habitat patches and links capture dispersal of organisms between these locations. Spatial network structure (topology) is key for understanding ecological patterns and processes such as the spread of perturbations (Gilarranz et al., 2017) and infections (Chinazzi et al., 2020; Davis et al., 2008); (Green et al., 2006) as well as range expansions (Rayfield et al., 2023) and the distribution of biodiversity in space (Carrara et al., 2012). Importantly, spatial network structure also drives evolutionary processes: Evolution is affected directly via gene flow within the network, but it can also be modulated indirectly via selection and drift due to the redistribution of population densities and genotypes within the spatial network (Fronhofer & Altermatt, 2017). Dispersal within the spatial network structure of landscapes is therefore a key eco-evolutionary driver (Fronhofer et al., 2023; Govaert et al., 2019).

Here, we focus on the spatial eco-evolutionary dynamics of one of the most pervasive interactions and, at the same time, maybe one of the most relevant to human and organismal health, more generally, parasitism. The spatial network structure of host–parasite systems is critical for understanding epidemiological and (co)-evolutionary dynamics (Parratt et al., 2016; Penczykowski et al., 2016; White et al., 2018). For example, increased spatial clustering has been shown to reduce epidemic spread (Lilley et al., 2018), spatial network heterogeneity may lower epidemic thresholds (Colizza & Vespignani, 2008) and landscape properties affect rates of disease spread along river networks (Carraro et al., 2018). Incorporating complex landscape structures can help predict occupancy and persistence of infection (Hess, 1996): Jousimo et al. (2014) have shown in the *Plantago* (plant)–*Podosphaera* (fungal) natural host–parasite system, that increased resistance likely driven by greater gene flow in *Plantago* at highly connected habitat patches, leads to reduced parasite occupancy, when compared with less well-connected patches, whereas from a purely ecological perspective higher dispersal should lead to greater parasite occupancy in more connected metapopulations (Hess, 1996).

While in epidemiological studies, the effect of structure of complex networks, e.g., those with heterogeneous degree distributions (Colizza & Vespignani, 2008), has been studied extensively, how host and parasite traits respond to network structure is unclear. Instead, in evolutionary epidemiology, simplified spatial structures, such as lattice contact networks (Boots & Sasaki, 1999; Lion & Boots, 2010) and homogeneous (Gandon & Michalakis, 2002) or spatially implicit island models (Wild et al., 2009), have been used to understand the mechanisms driving host–parasite (co)-evolution. Particularly, previous work (Messinger & Ostling, 2009) has focused on the impact of such simplified spatial structures on virulence, a key parasite life history trait that can evolve (Cressler et al., 2016; Frank, 1996) and impact host demography (Hudson et al., 1998; May & Anderson, 1978). Virulence is defined as the extent to which parasites harm their hosts either by reducing host survival (Anderson & May, 1982) or fecundity (Abbate et al., 2015; O’Keefe & Antonovics, 2002). Parasite virulence is assumed to be a by-product of the fact that parasites need to exploit their hosts to transmit more (virulence–transmission trade-off hypothesis; Alizon et al.^2009^; [Bibr CIT0004][Bibr CIT0004]). Models of simplified spatial structures have highlighted the evolutionary mechanisms, particularly, self shading (Boots & Sasaki, 1999) and kin selection (Lion & Boots, 2010; Wild et al., 2009), that drive the evolution of parasite virulence. Generally, spatial structure can limit the availability of resources (hosts) to parasites, and also lead to genetic structuring (relatedness patterns; Lion et al.^2011^) which can, in turn, lead to the evolution of reduced virulence as an altruistic strategy. Thus, while the role of spatial structure in driving the evolution of parasite virulence is widely recognized in general (Kerr et al., 2006; Messinger & Ostling, 2009), how realistically complex landscapes impact its evolution is not known.

As a proof of concept, we here consider the evolution of parasite virulence across biomes in characteristically terrestrial-like vs. river-like landscapes. Interestingly, spatial networks, whether terrestrial-like or river-like, have specific properties due to the physical processes that govern landscape genesis. Specifically, all riverine aquatic landscapes exhibit statistical properties that can be captured by optimal channel networks (OCNs) which are generated based on geomorphological processes that shape rivers globally (Carraro et al., 2020; Rinaldo et al., 2014). These networks are dendritic and and have many patches with one neighbor only, and few patches with multiple neighbors (highly heterogeneous degree distribution), which can impact, for example, metapopulation and metacommunity dynamics (Carrara et al., 2012). Similarly, terrestrial landscapes are intrinsically modular, that is, patches can be classified into modules: Patches are highly connected to each other within a module but sparsely connected to patches in other modules because of biological constraints on dispersal distances. This can be captured by random-geometric graphs (RGGs; Gilarranz, 2020). Modularity has consequences, for example, for the spread of perturbations in space (Gilarranz et al., 2017).

In this context, we develop an individual-based metapopulation model of host–parasite dynamics in which parasite virulence evolves. We assume that parasites rely on hosts for dispersal, host and parasite generations are discrete and non-overlapping. Furthermore, virulence acts by reducing host fecundity which is in contrast to a lot of previous work that focuses on the evolution of mortality virulence in simplified spatial structures (Lion & Boots, 2010). Spatial structure of the host–parasite system is modeled by networks representing terrestrial-like (RGGs) and river-like landscapes (OCNs). By analyzing this model, we (1) explore how spatial network topology, specifically that of terrestrial-like and river-like landscapes, impacts evolutionarily stable parasite virulence, (2) run additional simulation experiments demonstrating the role of kin selection in driving landscape-based evolutionarily stable virulence and outline the mechanisms by which spatial network topology generates characteristic patterns of parasite relatedness that drive these differences, and (3) by comparing with simulations in which parasite virulence is fixed, we analyze the ecological consequences, specifically parasite extinction probabilities, of landscape-specific virulence evolution.

## Methods

### Model overview

**Fig 1:**
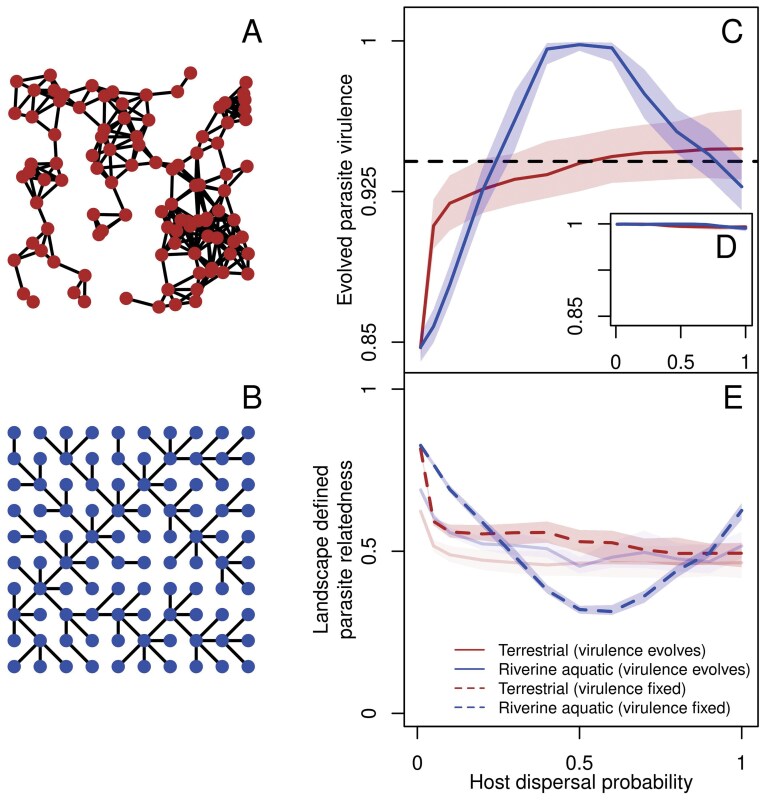
Terrestrial-like (A) and river-like landscapes (B) lead to differences in virulence evolution (C) which vanish when parasite kin structure is broken (D). Thus, differences in virulence evolution between the two landscape types is explained by diverging patterns of landscape defined parasite relatedness (E). (A) A representative terrestrial-like landscape modeled as an RGG. (B) A representative river-like landscape modeled as an OCN. (C) Evolved parasite virulence as a function of host dispersal in river-like (blue) and terrestrial-like (brown) landscapes. The solid line represents the median and shaded regions the inter-quartile range of the evolved virulence trait over simulations in 1,000 realizations of each landscape type, median over all infected individuals at the last simulation time step (t=2,500) for the simulations in which the parasite persists in the landscape. The dashed line indicates fixed virulence v=0.94 which is used to represent landscape defined parasite relatedness in (E). (D) Evolved parasite virulence as a function of host dispersal in terrestrial-like (brown) and river-like (blue) landscapes for simulations in which parasite kin structure is broken by shuffling parasites post infection, while maintaining the same infected and susceptible densities. The lines and shaded regions are the same as (C), except that we include simulations where the parasite goes extinct. (E) Average parasite relatedness as a function of host dispersal in terrestrial-like (brown) and river-like (blue) landscapes within a patch in ecological simulations with fixed virulence (dashed lines) and when virulence can evolve (faint solid lines). We plot the average parasite relatedness over the last 100 time steps within a patch over all patches over 100 replicates. Details of landscape generation and calculation of parasite relatedness are found in Methods and Supplementary material. Model parameters of the focal scenario: intrinsic growth rate of the host $\lambda_{0}=4$, intraspecific competition coefficient of the host α=0.01, maximum parasite transmission $\beta_{max}=8$, shape of trade-off curve s=0.5, searching efficiency of parasite a=0.01.

We developed an individual-based susceptible-infected (SI) metapopulation model in which parasite virulence is genetically encoded and can evolve. This model is adapted from the model developed by Chaianunporn and Hovestadt (2012). Hosts and parasites are asexual, haploid, and have discrete, non-overlapping generations. Host dispersal is natal, and hosts disperse to neighboring patches within their spatial network with a fixed probability (d). We assume that dispersal does not evolve, thus our model applies in cases where parasites evolve at a faster rate than hosts. Parasites rely on their hosts for dispersal. Intraspecific competition and parasite transmission are local. Virulence reduces the fecundity of the host and increasing virulence implies increasing transmission (Alizon et al., 2009; Anderson & May, 1982). We explore virulence evolution in terrestrial-like (represented by RGGs, Figure 1A) and in river-like landscapes (represented by OCNs, Figure 1B) of n=100 patches. A summarized description of the life cycle, landscapes, and analyses is given below and details are found in Supplementary material.

### Life cycle

#### Dispersal

After individuals are born, they disperse with probability d, to one of the patches connected to the natal patch with equal probability. The spatial network structure determines the connectivity of patches and is described below.

#### Reproduction

After dispersal, individuals reproduce. Local population growth follows Beverton–Holt dynamics (Beverton & Holt, 1957) with intrinsic growth rate $\lambda_{0}$ and intraspecific competition coefficient α:


$$\begin{array}{ccc} N_{x}(t+1)=\frac{\lambda_{0}S_{x}(t)}{1+\alpha N_{x}(t)}+\frac{\left(1-v)\lambda_{0}I_{x}(t\right)}{1+\alpha N_{x}(t)} & & \end{array}$$ (1)

where $S_{x}(t)$, $I_{x}(t)$, and $N_{x}(t)$ are the number of susceptible, infected, and total individuals in a patch x immediately post dispersal, $N_{x}(t+1)$ is the expected number of offspring, all of which are born susceptible. Virulence (v) acts on the fecundity of the host (Abbate et al., 2015; Jaenike, 1996; O’Keefe & Antonovics, 2002), so infected individuals produce 1−v times the number of offspring as susceptible individuals. The realized number of offspring is Poisson distributed. After reproduction, the parental generation dies and is replaced by the offspring generation.

#### Parasite transmission

Parasite propagules are then released from the dead infected individuals, which then find susceptible new-born hosts in the offspring generation according to a modified Nicholson–Bailey model (Equation 2) with a type II functional response (Chaianunporn and Hovestadt, 2012; Deshpande et al., 2021), in which the transmission rate is given by β(v) and the searching efficiency by a. Note that we assume that the propagules do not remain in the environment after transmission, making our model different from those assuming environmental transmission (Bonhoeffer et al., 1997; Kamo & Boots, 2004). Since the number of susceptible newborn hosts is $N_{x}(t+1)$, the expected number of infected hosts $I_{x}(t+1)$ is given by


$$\begin{array}{ccc} I_{x}(t+1)=N_{x}(t+1)\left(1-\exp\left(\frac{-a\beta (v)I_{x}(t)}{1+aN_{x}(t+1)}\right)\right). & & \end{array}$$ (2)

The transmission rate [β(v)] of parasites is assumed to increase with virulence (Equation 3; Alizon et al.^2009^) as


$$\begin{array}{ccc} \beta (v)=\beta_{\text{m}ax}v^{s}. & & \end{array}$$ (3)

Here, $\beta_{\text{m}ax}$ is the maximum possible transmission rate and s determines the shape of the virulence–transmission relationship, where 0<s<1, s=1, and s>1 represent saturating, linear, and accelerating relationships between virulence and transmission. Multiple parasite strains can find a host, but eventually only one can infect it. Virulence undergoes mutation with a probability $m_{v}$ and mutation effects on logit transformed virulence are drawn from a normal distribution with standard deviation $\sigma_{v}$. Parasites also inherit a neutral locus which is used to track relatedness between parasite strains.

### Landscapes

We model terrestrial-like and river-like landscapes using RGGs and OCNs, respectively. For each landscape type we generate 1,000 realizations of n=100 patches that are encoded in adjacency matrices. RGGs are generated using R package igraph (Csardi & Nepusz, 2006), version 3.6.3, by drawing n points from a U[0,1]×[0,1] distribution, and points that are within a radius r are neighbors (radius parameters of r=0.15; average degree 6.08 after discarding networks that are not connected Saade et al.^2023^). Increasing the radius would just lead to a metapopulation with global dispersal. OCNs are generated on un-aggregated 10×10 grid (Carraro et al., 2020) with a fixed outlet position. The average degree of OCNs is 1.98. We do not explicitly model riverine directionality as this has been shown to only make ecological patterns stronger (Fronhofer & Altermatt, 2017).

Additionally, since terrestrial-like and river-like landscapes are very different in their average degree, to identify the network properties that lead to differences in virulence evolution between landscape types, we model control landscapes which are described in detail in Supplementary material, Methods. Note that designing comparable terrestrial-like (RGG) and river-like landscapes (OCN) with the same average degree is not possible, since OCNs always have the same average degree based on the number of patches (1.98 for n=100 patches), given that they are spanning trees (Rinaldo et al., 2014). RGGs with this average degree will not be fully connected.

### Model analysis

Our main response variable is evolved parasite virulence which we measure at the the end of each simulation (at t=2,500, see Supplementary Figure S1) where evolutionary dynamics have reached an equilibrium. We run simulations for all parameter combinations in Supplementary Table S1, and the main text results are presented for one focal scenario that is described in all figure captions.

### Testing the role of kin selection

Previous work (Lion & Boots, 2010; O’Keefe & Antonovics, 2002; Wild et al., 2009) has argued that kin selection drives virulence evolution in spatially structured systems. This means that reduced virulence can evolve as an altruistic strategy under conditions of limited host availability when parasite relatedness is high.

#### Shuffling parasites to break kin structure

To test the role of kin selection in driving parasite virulence, we developed additional “shuffled” simulations in which after each transmission step, parasite genotypes are redistributed in the landscape while maintaining the same total host population size and infected densities in each patch. This breaks kin structure, without changing epidemiology (Deshpande et al., 2021; Poethke et al., 2007).

#### Measuring parasite relatedness

We further test whether patterns of parasite virulence are consistent with kin selection by running additional ecological controls in which virulence is fixed (v=0.8,0.82,...,1) to show how relatedness and host availability should change when virulence does not evolve. Running fixed virulence simulations allows us to identify the direction of causality since host availability and parasite relatedness both depend on virulence values but also drive its evolution. We measure parasite relatedness as:


$$\begin{array}{ccc} R_{x}=\underset{k}{\sum }p_{k,x}^{2} & & \end{array}$$ (4)

where $R_{x}$ is the probability of drawing two same neutral alleles in a patch x and $p_{k,x}$ is the frequency of an allele of type k in patch x (Rousset, 2004). We measure relatedness within a patch because this is the scale at which parasites interact with each other. The neutral locus mutates at the same rate as the virulence trait.

### Evaluating ecological consequences of evolved virulence

To understand how evolved virulence impacts the distribution of hosts and parasites in space, we compare the distribution of evolved virulence and several ecological variables between terrestrial-like and river-like landscapes. Specifically, we study the rate of parasite extinction, host densities, and prevalence as a function of host dispersal and patch degree for fixed values of virulence (v=0.8,0.82,...,1) and compare those results with simulation in which virulence can evolve.

## Results and discussion

### Landscape structure impacts the evolution of parasite virulence

#### Patterns of virulence evolution in terrestrial-like and river-like landscapes

We find that evolved parasite virulence differs qualitatively between terrestrial-like (Figure 1A) and river-like landscapes (Figure 1B) depending on host dispersal (Wild et al., 2009). For terrestrial-like landscapes, we observe a positive and saturating relationship between evolved virulence and dispersal (Figure 1C), where virulence is greatest at the highest dispersal values. In contrast, river-like landscapes produce a unimodal relationship, where virulence peaks at intermediate dispersal values and attains a greater maximum value (Figure 1C; for a more comprehensive analysis, see Supplementary Figures S2 and S3).

Additional analyses show that these landscape specific effects hold for greater maximum transmission ($\beta_{max}$ = 10) and for different shapes of the virulence–transmission relationship (saturating: *s* = 0.5; linear: *s* = 1; accelerating: s=2; Supplementary Figure S2). However, for lower transmission ($\beta_{\text{m}ax}=4$) and lower host population intrinsic growth rate ($\lambda_{0}=2$; Supplementary Figure S3) virulence evolution depends only on host dispersal and not on landscape structure. This is likely because, in our model, high levels of virulence and transmission lead to strong oscillations in host–parasite densities (Deshpande et al., 2021). Going out of this dynamical regime (see Supplementary Figure S4) reduces the differences between the two landscapes because lower amplitude oscillations do not induce local extinctions of the host. Thus, host availability is not limited and parasite kin selection becomes less important (see below).

#### Which network properties explain parasite virulence evolution?

**Fig 2:**
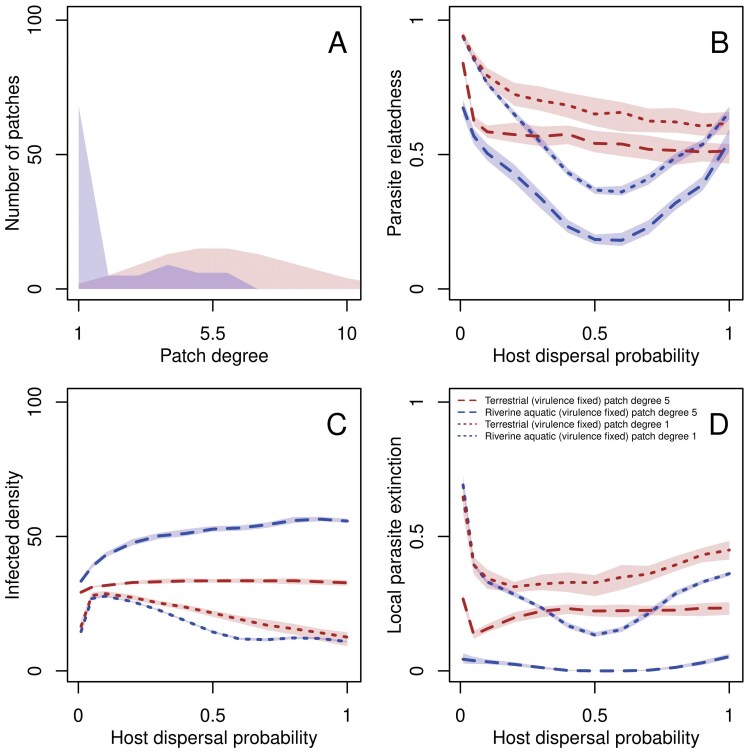
Degree distribution (A), parasite relatedness (B), infected densities (C), and local parasite extinctions (D) as a function of dispersal for low (degree-1) and high (degree-5) connectivity patches for river-like and terrestrial-like landscapes. (A) Degree distribution of terrestrial-like vs. river-like landscapes. The *x*-axis represents patch degree and the shaded region shows the median frequency of a patch of a given degree over 1,000 landscape realizations for both landscape types. (B) Parasite relatedness as function of dispersal rate for low- and high-degree patches. (C) Average density of infected individuals for low- and high-connectivity patches in the landscape as a function of host dispersal over the last 100 time steps. (D) Local parasite extinction calculated as the fraction of time over the last 100 time steps for which the parasite is extinct for low- and high-connectivity patches. For all plots, medians are taken over for 100 and 1,000 realizations for ecological and evolutionary simulations, respectively. Model parameters of the focal scenario: intrinsic growth rate of the host $\lambda_{0}=4$, intraspecific competition coefficient of the host α=0.01, maximum parasite transmission $\beta_{max}=8$, shape of trade-off curve s=0.5, searching efficiency of parasite a=0.01.

Since the processes that generate terrestrial-like (Gilarranz, 2020) and river-like (Rinaldo et al., 2014) landscapes are fundamentally different, these networks differ in a range of properties, including their average degree, that is, the average number of links between patches (see Figure 2A). Using a series of control landscapes where we can fix the average degree while varying other properties (see Supplementary material, Methods, and Supplementary Figures S5 and S6 for details) we show that the results presented in Figure 1C are not due to mere differences in the average number of links. Rather, virulence evolution in river-like landscapes is mainly driven by heterogeneity, or the presence of large numbers of headwaters (degree-1 patches), that is, patches that are only connected to one other patch (Figure 2A; Supplementary Figure S5). This is supported by the observation that the unimodal shape of virulence with dispersal is not recaptured by regular circular networks which have a similar average degree but rather by “spiky” networks, which have a similar average degree and a heterogeneous degree distribution (Supplementary Figure S5). This key role of spatial heterogeneity is elaborated below. Our results for terrestrial-like landscapes critically depend on networks being spatially embedded (as opposed to networks such as random graphs which do not have a spatial generating process) and, to a lesser degree, being modular (Supplementary Figure S6).

### Landscape structure drives parasite virulence evolution via kin selection

Clearly, network topology is an important driver of parasite virulence evolution. But what are the relevant evolutionary mechanisms and how are they modulated by landscape structure?

#### Kin selection drives differences in virulence evolution between terrestrial-like andriver-like landscapes

We show in Figure 1D that when kin structure is broken by shuffling parasites (Deshpande et al., 2021; Poethke et al., 2007) post-transmission, virulence evolves to its maximal value (v=1) in both terrestrial-like and river-like landscapes. Thus, differences in virulence evolution between the two landscape types are driven by inclusive rather than direct fitness effects (Poethke et al., 2007). Our result that virulence evolves to its maximal value in both landscape types matches the theoretical expectation from previous work modeling virulence acting on host fecundity (O’Keefe & Antonovics, 2002). More specifically, maximal virulence evolves in both landscape types when kin structure is broken because increase in virulence leads to increased transmission, without an individual cost to parasite fitness which is a feature of models of virulence acting on fecundity (O’Keefe & Antonovics, 2002).

However, when kin structure is intact (Figure 1C), locally parasites are related to each other (that is, they share identical alleles), and under conditions of limited host availability (Supplementary Figure S7) which in our model are induced by oscillations (see Supplementary Figure S4), can select for lower virulence relative to the expectation from mean field (O’Keefe & Antonovics, 2002), and more importantly depend on landscape structure (Figure 1C). Thus, our result is consistent previous work on evolution of virulence acting on host fecundity (O’Keefe & Antonovics, 2002) and mortality (Lion & Boots, 2010; Wild et al., 2009) that highlights the central role of kin selection in driving parasite virulence evolution in spatially structured host–parasite systems. Below, we show how landscape structure and host dispersal modulate parasite relatedness.

#### Patterns of virulence evolution mirror within-patch relatedness in terrestrial-likevs. river-like landscapes

Therefore, since differences in virulence evolution between the two landscape types are driven by kin selection, we would expect that more virulent parasites evolve when local parasite relatedness is low and vice versa, given that host availability is similarly limited (Lion & Boots, 2010) between the two landscape types (Supplementary Figure S7). Indeed, Figure 1E shows that river-like landscapes produce a U-shaped relationship between relatedness and dispersal attaining a minimum relatedness lower than terrestrial-like landscapes at intermediate dispersal rates, whereas terrestrial-like landscapes produce a saturating decrease. These patterns exactly mirror the relationship between dispersal and evolved virulence (Figure 1C). Note that parasite relatedness and host availability are measured in simulations in which virulence is fixed (v=0.94 in the main text, a wider range of virulence values is found in Supplementary Figure S7) since virulence both impacts relatedness (Supplementary Figure S7) and is driven by it (Figure 1D). The mirroring of parasite relatedness and evolved virulence also holds for all control landscapes (Supplementary Figures S8 and S9). Thus, ultimately, differences in how landscapes structure genotypes in space differently, as captured by parasite relatedness (Figure 1E), drives differences in virulence evolution (Figure 1D). Below, we explain the spatial network properties that lead to differences in parasite relatedness, hence virulence evolution between terrestrial-like and river-like landscapes.

#### Spatial network properties determining parasite relatedness

We now seek to understand the network properties that drive differences in within-patch relatedness between terrestrial-like and river-like landscapes. As shown above, the shape of the degree distribution is relevant, particularly the large amount of degree-1 patches in river-like landscapes (Figure 2A). For both landscapes, parasite relatedness is low in patches with high connectivity and high in patches with low connectivity (Figure 2B). Furthermore, at lower dispersal rates (up to d=0.1), local connectivity dominates and all landscapes have similar distributions of parasite relatedness given a patch degree. But since there are many more degree-1 patches in river-like relative to terrestrial-like landscapes (Figure 2A), relatedness averaged over all patches in the landscape is greater in river-like landscapes. As dispersal increases, due to the heterogeneity in degree distribution in river-like networks (specifically, many patches of degree-1 connected to few patches of larger degree and few patches of a larger degree connected to many patches of degree-1; Figure 2A), there is greater dispersal out of low-connectivity patches and lower dispersal into them (Supplementary Figures S10 and S11; [Bibr CIT0023][Bibr CIT0023]; [Bibr CIT0003][Bibr CIT0003]), which leads to relatively lower infected densities in low degree patches and larger infected densities in high degree patches relative to terrestrial-like networks (Figure 2C). This implies that the parasite rarely goes extinct in high degree patches and recolonizes low-degree patches of river-like landscapes, reducing the effect of genetic drift in the landscape and leading to lower relatedness relative to terrestrial-like landscapes (Figure 2D). Finally, as dispersal increases further, infected densities are further reduced in low-degree patches, which leads to increasing extinction in high-degree patches, and overall greater relatedness.

These analyses of neutral genetic diversity provide a mechanistic explanation of how landscape network structure shapes parasite relatedness, through the specific patterns of patch connectivity and the concomitant dynamics of patch extinction and (re)-colonization. The resulting differences in parasite relatedness between river-like and terrestrial-like landscapes are perfectly in line with the hypothesis that virulence evolution is driven by kin selection.

### Closing the eco-evolutionary feedbacks loop: ecological consequences of virulenceevolution in river-like and terrestrial-like systems

**Fig 3:**
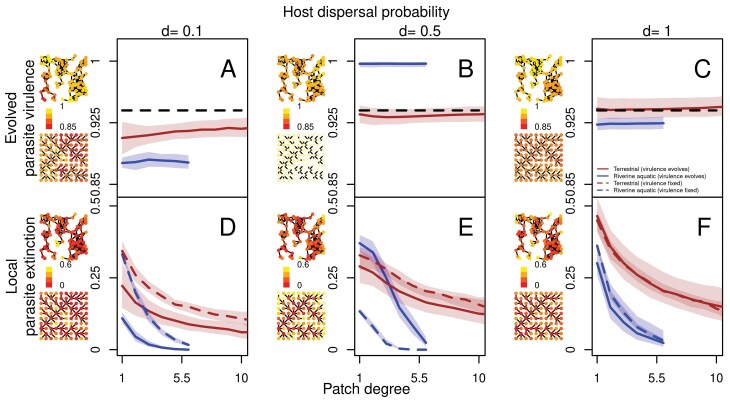
Spatial distribution of evolved parasite virulence (A–C) and local parasite extinction (D–F) as a function of patch degree for terrestrial-like (brown) and river-like (blue) landscapes for different host dispersal probabilities (d=0.1,0.5,1) in ecological (virulence v=0.94, dashed line) and evolutionary (solid lines) simulations. From left to right, dispersal probability increases. (A–C) Evolved parasite virulence in a patch, averaged over the last 100 time steps is plotted as a function of patch degree. On the left-hand side of each plot, the distribution of evolved virulence averaged over the last 100 time steps for one representative terrestrial-like and river-like landscape is shown with more yellow colors indicating higher virulence. (D–F) Local parasite extinction calculated as the average fraction of time in which parasite is absent from a patch is plotted as a function of patch degree. On the left-hand side of each plot, the distribution of local parasite extinction over the last 100 time steps for one representative terrestrial-like and river-like landscape is shown with more yellow colors indicating higher local parasite extinction. For all plots, medians are taken over for 100 and 1,000 realizations for ecological and evolutionary simulations, respectively. Model parameters of the focal scenario: intrinsic growth rate of the host $\lambda_{0}=4$, intraspecific competition coefficient of the host α=0.01, maximum parasite transmission $\beta_{max}=8$, shape of trade-off curve s=0.5, searching efficiency of parasite a=0.01.

While the above sections worked out the mechanisms driving virulence evolution, here we ask how evolved parasite virulence feeds back on spatial epidemiological dynamics. We focus on a key epidemiological variable, local parasite extinction, i.e., the fraction of time there is no parasite in a given patch. As shown above (Figure 2D), for purely ecological scenarios, there is characteristic variation in extinction risk according to network type. Below, we will assess the additional impact resulting from virulence evolution, for different levels of host dispersal. As a general rule, except for very low dispersal rates (see Supplementary Figures S12 and S13), virulence is independent of patch connectivity (degree; Figure 3A–C). Thus, differences in virulence evolution between landscape types (Figure 1C), and not within a landscape (Figure 3A–C) should explain differences in local parasite extinction patterns that will be described below.

Without accounting for differences in parasite virulence in both landscape types, thus in simulations in which virulence is fixed (v=0.94 in the main text for illustrative purposes; see Supplementary Figures S12 and S13 for a wider range of virulence values), terrestrial-like and river-like landscapes produce distinct relationships between patch connectivity and local parasite extinction probability (Figure 3D–F). Depending on the level of evolved virulence, extinction risk is shifted toward higher or lower levels, relative to this ecological reference scenario of fixed virulence (Figure 3D–F).

At relatively low dispersal rates (d=0.1; Figure 3D), from a purely ecological perspective, low connectivity patches have similar high local parasite extinction for a fixed equal virulence in terrestrial-like and river-like landscapes. But evolution of lower virulence in river-like landscapes leads to reduced local parasite extinction irrespective of connectivity relative to terrestrial-like landscapes. At intermediate dispersal (d=0.5; Figure 3E), without accounting for differences in virulence evolution, one would predict lower parasite extinction as a function of connectivity throughout the landscape in river-like relative to terrestrial-like landscapes due to the heterogeneity in degree distribution (Figure 2A) maintaining high-infected densities (Figure 2C) such that all patches in the landscape are always recolonized. However, the evolution of greater virulence in river-like landscapes means that local parasite extinction is higher in low-connectivity patches, and lower in high-connectivity patches in river-like landscapes relative to terrestrial-like landscapes. Finally, at high dispersal (d=1; Figure 3F), since virulence evolves to similar values in both landscape types, assuming similar virulence, one can predict accurately spatial distribution.

These results illustrate how knowing evolutionary optima for parasite virulence can allow us to make general predictions about differences in spatial parasite distribution across landscape types. Thus, overall, by accounting for evolutionary stable virulence, we show that we should find greater local parasite extinction in river-like landscapes (Supplementary Figure S14). Interestingly, since most of these extinctions are located in degree-1 patches (Figure 3D–F), this decreases the likelihood that the parasite goes extinct globally, relative to terrestrial-like landscapes in which local extinctions are more are less variable across connectivity. Other examples, namely parasite prevalence and host population densities (Supplementary Figure S15) show that differences in virulence evolution between the two landscape types do not change predictions relative to what is expected for a fixed virulence.

Thus, with these examples, we highlight the complex interplay between landscape structure, evolutionarily stable virulence and predicting epidemiological variables.

## General discussion

In summary, we find that virulence acting on fecundity evolves differently in terrestrial-like and river-like landscapes, with kin selection as the main evolutionary driver. Furthermore, by analyzing genetic structure we show that the two landscapes produce characteristic patterns of parasite relatedness that explain differences in virulence evolution. We further show how network topology, through the specific patterns of patch connectivity and their impact on demography, generates differences in parasite relatedness between terrestrial-like and river-like landscapes. Ultimately, the interplay between network-specific ecological effects and network-specific evolutionarily stable virulence defines the spatial distribution of hosts and parasites, and more broadly host-parasite metapopulation dynamics.

**Fig 4:**
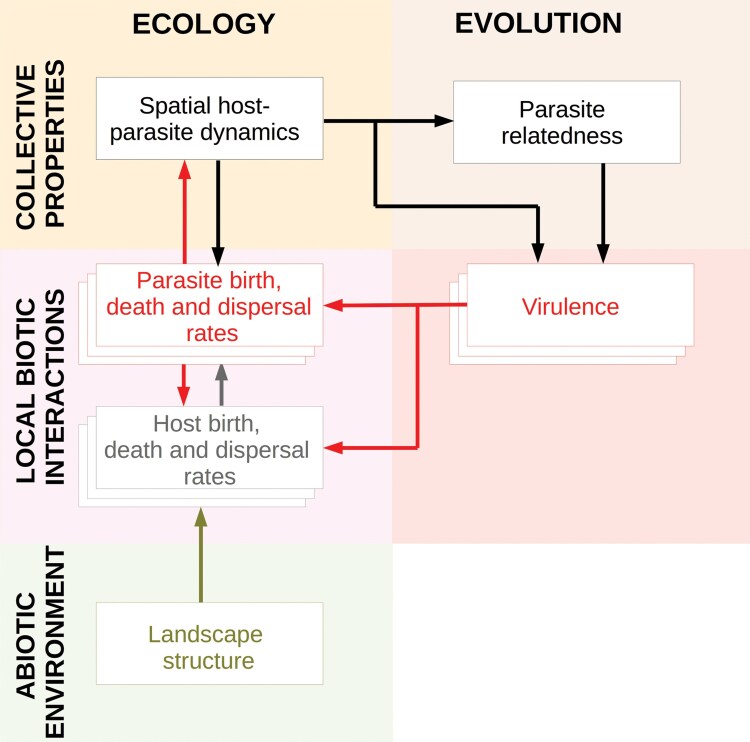
An eco-evolutionary feedback between host–parasite demography, landscape structure and virulence evolution. Clearly, landscape structure (green box) represents the abiotic environment, the biotic host–parasite interactions are shown by stacked boxes of hosts (gray) and parasites (red), ecological interactions and evolutionary dynamics represented by the virulence genotypes (red). Together, the abiotic environment, demography and evolution lead to collective properties (indicated by black boxes) at a higher level of organization that can further feed back onto demographic rates. The color of the arrows indicates the boxes from which they originate. In our study, the landscape context is the abiotic environment that impacts host and parasite dispersal, since it impacts host (hence, parasite) dispersal patterns, and also their demography by modifying their spatial distribution. Virulence is genetically encoded, and it directly impacts parasite demography, via the virulence–transmission trade-off, and host demography by reducing the number of offspring the host produces (evo-to-eco). Finally, patterns of host availability and parasite relatedness that result from host–parasite demography feed back onto parasite demographic rates, which, via selection and gene flow, impact trait evolution (eco-to-evo).

Our modeling exercise allows us to identify a mechanistic eco-evolutionary feedback loop (Figure 4; Fronhofer et al.^2023^; Govaert et al.^2019^). Ecologically, landscape structure impacts dispersal patterns of individual hosts and their parasites, leading collectively to characteristic host-parasite spatial dynamics. These spatial network specific dynamics further define patterns of parasite relatedness in the landscape, which impacts the evolution of parasite virulence (eco-to-evo). Parasite virulence evolution in turn impacts the demographic rates of the hosts and parasites driving ecological patterns at the level of the patch (e.g., patterns of local parasite extinction), but also the distribution of hosts and parasites at the landscape scale (evo-to-eco).

More generally, this feedback loop allows us to derive the empirically testable prediction that parasite virulence as a function of host dispersal should differ generally between terrestrial-like and river-like landscapes (Figure 1). This prediction remains untested despite recent efforts to quantify impacts of parasites on host reproduction (Hasik & Siepielski, 2022) in a meta-analysis since data are mostly available on terrestrial systems. Of course, natural host–parasite systems likely also have other drivers of virulence than landscape structure. Additionally, riverine aquatic and terrestrial parasites could differ generally in their life cycles. While we recognize these limitations, our model shows that, all else being equal, the landscape context alone can modify eco-evolutionary outcomes. Below, following the framework developed in Figure 4, we will discuss our work in the context of previous studies on virulence evolution, ecological dynamics in complex networks and eco-evolutionary feedbacks in host–parasite systems.

### Eco-to-evo: parasite relatedness, hence, strength of kin selection results fromnetwork topology

Focusing the eco-to-evo component of Figure 4, we emphasize the role of kin selection for virulence evolution in spatially structured systems. It has previously been shown in infinite island models (Wild et al., 2009) and using pair approximations in lattice models (Lion & Boots, 2010) that virulence evolution in spatially structured systems should be driven by kin selection. In Wild et al. (2009), increasing parasite dispersal leads to greater virulence due to reduced competition with kin locally (kin shading). Thus, evolution of reduced virulence in spatially structured systems can be thought of as an altruistic strategy (Lion & Boots, 2010), since it leads to a individual cost of reduced transmission and and inclusive fitness benefit of increased availability of susceptible hosts. Generally, the simplifying assumptions on spatial structure (Lion & Boots, 2010; Wild et al., 2009) allow for an analytical expression for selection gradients to be obtained. Thus, the mechanisms driving evolution of parasite virulence going from well mixed to simplified systems are well worked out. While modeling more complex spatial structure does not allow for analytical treatment, it is possible to partition individual and relatedness-based effects by re-shuffling simulations (Deshpande et al., 2021; Poethke et al., 2007), like in the present study, confirming the role of kin selection. Placed within this context, we show that parasite relatedness, hence the strength of kin selection itself results from spatial network topology, which determine local parasite relatedness, further driving the evolution of parasite virulence.

Some of the assumptions in our model differ from these studies, most notably we assume virulence acts on the fecundity of the host rather than mortality (Abbate et al., 2015). While fecundity virulence is found in many biological systems (Abbate et al., 2015), it is not modeled as frequently. The goal of our study was not to fill this gap, but rather to explore the mechanisms driving spatial network-based differences in trait evolution, with the evolution of fecundity virulence as an example. In our model, increased virulence, hence transmission, reduces the availability of susceptible hosts. When relatedness is high, this leads to a substantial inclusive fitness cost and can select against virulence acting on fecundity. With this mechanistic understanding in mind, insights from our model can be used to understand the evolution of other traits, such as virulence acting on mortality. For example, we speculate that qualitative differences between terrestrial-like and river-like landscapes should also apply to mortality virulence as long as the demographic feedback from increased transmission is high (e.g., when a highly castrating parasite is assumed), since this generates the characteristic patterns of parasite relatedness and host availability we find in our model. This is because, as our results indicate, the mechanism of kin selection that drives landscape-based differences in virulence evolution, acting on either fecundity and mortality, relative to a mean field model should be the same in spatially structured populations. Other assumptions that may impact the results are that of host–parasite co-dispersal and non-overlapping generations. Unlike our model, if parasites disperse independently of their hosts, we expect parasite virulence to depend on the spatial network along which parasites disperse and their dispersal rates, since parasite dispersal will drive the extinction-recolonization dynamics yielding characteristic patterns of parasite relatedness in terrestrial-like and river-like landscapes seen in Figure 2. The degree of overlap in host generations will possibly reduce the effect of landscape structure since constraints on host availability may not be as severe.

While it is now recognized that terrestrial and riverine landscapes have different properties (Carraro et al., 2020; Gilarranz, 2020; Rinaldo et al., 2014), we show that these properties lead to different evolutionary dynamics. Previous work has investigated the impact of modularity, which is a feature of terrestrial landscapes, and how it slows the spread of spatial perturbations (Gilarranz et al., 2017). Studies on river-like networks have focused theoretically and experimentally on metapopulation (Fronhofer & Altermatt, 2017) and metacommunity (Carrara et al., 2012) dynamics. Abstract representations of river-like landscapes have also been used in a more applied perspective to show differences in parasite spread upstream and downstream such landscapes (Carraro et al., 2018). While these studies focus only on one landscape type, Saade et al. (2023) have recently studied bistabilty and hysteresis comparing terrestrial-like and river-like metapopulations. Going beyond this, we show that not only should ecological dynamics differ between terrestrial-like and river-like landscapes, but also patterns of selection and gene flow, leading to differences in trait evolution. Therefore, the mechanism of kin selection reported here is only one example of how landscape structure impacts eco-evolutionary feedbacks. Specifically in our study, by generating an expectation of parasite relatedness that depends on landscape structure and dispersal, but not on evolved virulence, we can investigate the impact of ecology (landscape structure) on patterns of parasite relatedness hence, virulence evolution by breaking the feedback of evolution on ecology.

Our result, that is, it is the heterogeneity in connectivity of river-like networks that leads to characteristic patterns of parasite relatedness, can be placed in the context of previous work on patterns of neutral genetic and species diversity in spatial networks. Boussange and Pellissier (2022) study this effect of network structure on neutral genetic diversity and its consequences on evolution of local adaptation to an environment. They show that neutral genetic differentiation in spatial networks depends on both connectivity and variation in population densities due to heterogeneous degree distributions. Similarly, it has been shown theoretically that river-like networks maintain greater genetic diversity compared with grid landscapes (Paz-Vinas & Blanchet, 2015; Thomaz et al., 2016). Parallels can also be drawn to characteristic patterns of species diversity in spatial networks (Economo & Keitt, 2008) and particularly riverine networks (Carrara et al., 2012; Muneepeerakul et al., 2007). Finally, spatial network structure has also been shown to drive the evolution of dispersal (Fronhofer & Altermatt, 2017; Henriques-Silva et al., 2015; Muneepeerakul et al., 2011).

Importantly, while going from a simplified spatial context (such as infinite island models), to more complex spatial structures, the basic mechanisms driving trait evolution do not change. However, landscape structure acts to modulates them (Figure 4). In a host–parasite context, we here study an illustrative example of virulence evolution; however, patterns of local adaptation as a result of co-evolution also critically depend on gene flow (Gandon & Michalakis, 2002) within networks (Gibert et al., 2013; Höckerstedt et al., 2022; Jousimo et al., 2014). Beyond host–parasite systems, linking our results to kin selection also implies that landscape structure should modulate the evolution of social behavior, in general.

### Evo-to-eco: differences in virulence evolution may amplify, override or reducedifferences between terrestrial-like and river-like landscapes at different spatialscales

Finally, taking into account how virulence evolution differs between terrestrial-like and river-like landscapes, we can predict the spatial distribution of hosts and parasites. As discussed above, even without differences in virulence evolution, terrestrial-like and river-like landscapes are generally expected to differ in their ecological properties (see, for example, Saade et al.^2023^). Figure 3 re-emphasizes this point, where the fixed virulence scenario shows differences in the spatial distribution of local parasite extinction between terrestrial-like and river-like landscapes. First, this shows that ecological variables as a function of patch degree can only be understood in the context of the entire landscape, since for the same connectivity they differ between two landscape types. Second, taking an evolutionary perspective allows us to understand how differences in trait evolution can increase, decrease or not impact ecological variables given similar connectivity in different landscapes. Thus, accurate predictions of disease distributions and spread may require information on landscape topology. Our work demonstrates how virulence evolution can in turn impact host–parasite ecological dynamics (e.g., patterns of local parasite extinction) at different spatial scales (evo-to-eco). Considering virulence evolution in terrestrial-like and river-like landscape allows us to show how differences in the spatial distribution of hosts and parasites between such landscapes can be reduced (in the case of local parasite extinction, and intermediate dispersal), amplified (in the case of local parasite extinction, and low dispersal) or stay the same (host population density and parasite prevalence for all dispersal rates), as a consequence of virulence evolution. This complex interaction can be explained based on Figure 4, since landscape structure directly impacts the spatial distribution of hosts and parasites. Because this in turn impacts how virulence evolves, the consequences of virulence evolution are indirect, and differences in landscape structure alone may dominate. At the level of the metapopulation, landscape structure clearly dominates over differences in evolved virulence for parasite persistence (global parasite extinction), with global extinctions being greater for terrestrial-like landscapes even though evolved virulence is lower. Thus, the interaction between landscape type and evolution must be considered when trying to predict spatial epidemiology. Recent work by Jousimo et al. (2014) and Höckerstedt et al. (2022) support this finding, since the authors show that increased host-gene flow in highly connectivity patches leads to increased resistance, reducing harm to the host, and lower parasite occupancy.

### Future direction

While our study highlights how taking into account spatial network structure impacts our understanding of eco-evolutionary feedbacks in host–parasite systems, important questions remain to be answered. For example, in our study, we assumed that dispersal is fixed and cannot evolve, implying that our model applies to host–parasite systems in which the parasite evolves much faster than the host as is likely in host–parasite systems where the number of parasites is much greater than the host. However, host dispersal may also evolve in other kinds of systems and more importantly can be driven by landscape structure (Fronhofer & Altermatt, 2017; Henriques-Silva et al., 2015; Muneepeerakul et al., 2011) which can be explored in detail in further work. We also do not account for riverine directionality as this makes ecological and evolutionary patterns even stronger (Fronhofer & Altermatt, 2017), but a detailed investigation of more realistic river-like landscapes is possible.

### Conclusion

By studying how parasite virulence evolves in terrestrial-like and river-like landscapes, we highlight an eco-evolutionary feedback between landscape structure, host–parasite demography and parasite virulence (Figure 4). It is now increasingly recognized that eco-evolutionary dynamics of host–parasite systems (Rogalski et al., 2017) are greatly modified by anthropogenic change leading to phenomena such as emerging infectious diseases (Daszak et al., 2000). Particularly, landscapes are vulnerable to changes such as habitat fragmentation and increased barriers to dispersal, generally, rewiring of dispersal networks (Bullock et al., 2018). Such disturbances may be intrinsic (due to landscape structure) or extrinsic (due to the nature of human activities), and can differ between terrestrial and riverine systems (Johnson & Paull, 2011), thus requiring differing management strategies. Our work highlights that an eco-evolutionary perspective may be needed for a complete understanding and an efficient management of natural systems.

## Supplementary material

Supplementary material is available online at *Evolution Letters*.

## Supplementary Material

qraf003_suppl_Supplementary_Material

## Data Availability

Model code is available at via GitHub and Zenodo (https://zenodo.org/doi/10.5281/zenodo.10037324).
